# Comparative Effectiveness of Biosimilar, Reference Product and Other Erythropoiesis-Stimulating Agents (ESAs) Still Covered by Patent in Chronic Kidney Disease and Cancer Patients: An Italian Population-Based Study

**DOI:** 10.1371/journal.pone.0155805

**Published:** 2016-05-17

**Authors:** Ylenia Ingrasciotta, Francesco Giorgianni, Ilaria Marcianò, Jenny Bolcato, Roberta Pirolo, Alessandro Chinellato, Valentina Ientile, Domenico Santoro, Armando A. Genazzani, Angela Alibrandi, Andrea Fontana, Achille P. Caputi, Gianluca Trifirò

**Affiliations:** 1 Unit of Clinical Pharmacology, A.O.U. Policlinico ‘‘G. Martino”, Messina, Italy; 2 Treviso Local Health Unit, Treviso, Italy; 3 Department of Clinical and Experimental Medicine, AOU Policlinico “G. Martino”, University of Messina, Messina, Italy; 4 Department of Pharmaceutical Sciences, ''A. Avogadro'' University, Novara, Italy; 5 Department of Economic Sciences, University of Messina, Messina, Italy; 6 Unit of Biostatistics, IRCCS ‘‘Casa Sollievo della Sofferenza”, San Giovanni Rotondo, FG, Italy; 7 Department of Biomedical and Dental Sciences and Morpho-functional Imaging, University of Messina, Messina, Italy; 8 Department of Medical Informatics, Erasmus Medical Center, Rotterdam, Netherlands; FEDERICO II UNIVERSITY OF NAPLES, ITALY

## Abstract

**Background:**

Since 2007 biosimilars of erythropoiesis-stimulating agents (ESAs) are available on the Italian market. Very limited post-marketing data exist on the comparative effectiveness of biosimilar and originator ESAs.

**Aim:**

This population-based study was aimed to compare the effects of biosimilars, reference product and other ESAs still covered by patent on hemoglobinemia in chronic kidney disease (CKD) and cancer patients in a Local Health Unit (LHU) from Northern Italy.

**Methods:**

A retrospective cohort study was conducted during the years 2009–2014 using data from Treviso LHU administrative database. Incident ESA users (no ESA dispensing within 6 months prior to treatment start, i.e. index date (ID)) with at least one hemoglobin measurement within one month prior to ID (baseline Hb value) and another measurement between 2^nd^ and 3^rd^ month after ID (follow-up Hb value) were identified. The strength of the consumption (as total number of defined daily dose (DDD) dispensed during the follow-up divided by days of follow-up) and the difference between follow-up and baseline Hb values [delta Hb (ΔHb)] were evaluated. Based on Hb changes, ESA users were classified as non-responders (ΔHb≤0 g/dl), responders (0<ΔHb≤2 g/dl), and highly responders (ΔHb>2 g/dl). A multivariate ordinal logistic regression model to identify predictors for responsiveness to treatment was performed. All analyses were stratified by indication for use and type of dispensed ESA at ID.

**Results:**

Overall, 1,003 incident ESA users (reference product: 252, 25.1%; other ESAs covered by patent: 303, 30.2%; biosimilars: 448, 44.7%) with CKD or cancer were eligible for the study. No statistically significant difference in the amount of dose dispensed during the follow-up among biosimilars, reference product and other ESAs covered by patent was found in both CKD and cancer. After three months from treatment start, all ESAs increased Hb values on average by 2g/dl. No differences in ΔHb as well as in frequency of non-responders, responders and highly responders among different types of ESAs were observed in both indications of use. Overall, around 15–20% of ESA users were non-responders. Strength of treatment, but no type of dispensed ESAs was found to be predictor of responsiveness to treatment.

**Conclusions:**

No difference on the effects on hemoglobinemia among users of either biosimilars or reference product or ESAs covered by patent was observed in a general population from Northern Italy, despite a comparable dispensed dose of the different ESAs during the first three months of treatment.

## Introduction

Erythropoiesis-stimulating agents (ESAs) are biological analogues of human erythropoietin that are produced by cell lines throughout recombinant DNA technology. The main indications for use of ESAs are the treatment of anemia associated to chronic kidney disease (CKD) or chemotherapy-induced in cancer patients [1].

ESAs are indicated when hemoglobin (Hb) values are less than 11 g/dl in CKD patients and 10g/dl in the treatment of chemotherapy-induced anemia. In both indications, hemoglobinemia has to be maintained between 11 and 12g/dl [2], avoiding a rise in Hb greater than 2 g/dl over a four week period.

To date, seven ESA medicinal products (i. e. epoetin alfa, beta, zeta, theta, darbepoetin alfa, methoxypolyethyleneglycol-epoetin beta) are available on the Italian market.

Since 2007, epoetin alfa is one of a few biologics (in addition to filgrastim, somatropin, and, more recently, follitropin alfa, infliximab, and glargine insulin) for which biosimilars are currently available in Europe, and other biosimilars will be introduced in the European market in the near future (e.g. etanercept, pegfilgrastim, and trastuzumab). To date, US Food and Drug Administration (FDA) approved a biosimilar of filgrastim for all indications included in the reference product’s label.

As relevant differences in the acquisition price between biosimilar and reference product ranging from 15% to 30% have been documented [3–5], biosimilars represent an opportunity for saving healthcare resources to be reallocated to innovative medicines [3].

Regulatory agencies define a biosimilar (follow-on biologic in US) as ‘‘a biological medicinal product that contains a version of the active substance of an already authorized original biological medicinal product (reference medicinal product)”. In Europe, biosimilars are centrally approved by European Medicine Agency (EMA) based on “comprehensive comparability exercise” which has to prove similarity of biosimilar to the reference product in terms of quality characteristics, biological activity, safety and efficacy [6]. Nevertheless, biosimilars were not immediately well perceived, especially in the first years after their marketing, as demonstrated by the very low penetration of these drugs in most of the European Countries [7, 8].

Four pre-marketing clinical trials of biosimilar epoetin alfa were carried out on patients with CKD-related anemia showing no differences between biosimilar and reference product in achieving the Hb value target after 12 [9], 24 [10], 28 [11], or 56 [12] weeks of treatment. In addition, Palmer SC et al. performed a recent meta-analysis of available randomized controlled trials comparing the efficacy and safety of ESAs in patients with CKD, highlighting the lack of sufficient evidence that might suggest the superiority of any ESA formulation [13]. Only one double-blind, randomized, multicentre study was specifically carried out to assess the efficacy and safety after 12 weeks of treatment with a biosimilar epoetin alfa in treating chemotherapy-associated symptomatic anemia in patients with solid tumors [14]. On average, less than 500 patients were included in each premarketing randomized control study of biosimilar ESAs.

Taking into account evidence from post-marketing data, several observational studies proved the effectiveness of epoetin zeta [15], darbepoetin alfa [16], or biosimilar epoetin alfa [17] using real world data but without any comparator. Only one European multicenter retrospective study showed no difference about real-life clinical effectiveness and safety of biosimilar epoetin alfa vs. darbepoetin alfa for 4–5 weeks considering 429 patients with chemotherapy-induced anemia [18].

To further reassure about the comparability of effectiveness between biosimilar and originator ESAs, the aim of this observational, population-based study was to evaluate and compare the effects of biosimilar, reference product and other ESAs still covered by patent on haemoglobinemia in chronic kidney disease (CKD) and cancer patients, separately, in a Local Health Unit (LHU) from Northern Italy.

## Material and Methods

### Data Source

This was an observational, population-based, retrospective cohort study which is part of the Italian Health Ministry funded project “Assessment of short and long term risk-benefit profile of biologics through healthcare database network in Italy” (RF-2010.2320172) [8]. Fully anonymized data were extracted from the administrative databases of Treviso LHU, covering a total population of around 460,000 persons during the years 2009–2014. For each ESA prescription, specialists have to fill an electronic therapeutic plan reporting exact drug name, number of dispensed packages, dosing regimen and indication for use. This data can be linked through anonymized patient unique identifier to other claims databases containing data on drug dispensing, causes of hospitalization, healthcare service payment exemptions, outpatient diagnostic tests, values of laboratory tests, etc.

Drug dispensing is coded by Anatomical Therapeutic Chemical (ATC) classification system and the Italian marketing authorization code (AIC), which allows distinction between biosimilar, reference product and other ESAs still covered by patent, while indication for use and causes of hospitalizations are coded by International Classification of Disease, 9th revision, clinical modification (ICD9-CM). Additional details about data source can be found elsewhere [8].

### Study Population

**Fig 1:**
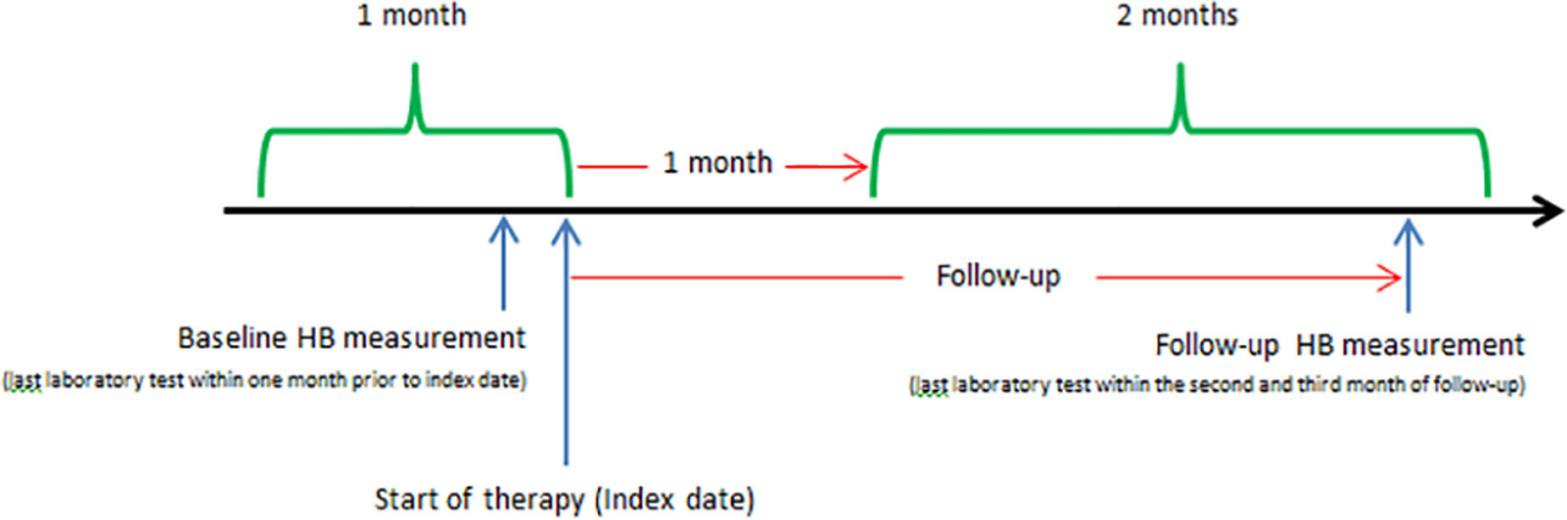
Depiction of follow-up period and Hb measurements. Hb = Hemoglobin.

Persons from the general population of Treviso LHU in the years 2009–2014 were included in the study based on the following criteria:

1. at least one year of database history;
2. at least one ESA dispensing during the study period with no drug dispensing within the six months prior (i.e. incident ESA users);
3. at least one hemoglobin measurement within one month prior to the date of the first ESA dispensing (i.e. Index Date-ID), defined as baseline Hb value, and another measurement between the 2^nd^ and the 3^rd^ month after ID (follow-up Hb value) (Fig 1). The same patient could be included multiple times in the study in case of withdrawal of ESA treatment for at least six months and the beginning of a new ESA treatment in the following period.

According to the Italian Medicines Agency, the term naïve patient refers to two specific categories: i) patients with no previous therapeutic exposure to originator (“primary naïve”); and ii) patients with previous exposure to the originator but with a wash-out period of time adequately long based on the judgment of the clinician (“secondary naïve”) [19]. So far there is no consent about secondary naïve definition for epoetin users. Based on previous evidence suggesting 5-fold half-life of epoetin as carry-over period, a 6 months wash out interval was considered as a reasonable time for identification of naïve users [20].

All analyses were stratified by indication for use and type of dispensed ESA at ID.

### Study Drugs

The use of the following ESAs during the study period was assessed: epoetin alfa (ATC: B03XA01; Eprex^®^, Abseamed^®^, Binocrit^®^), epoetin beta (B03XA01; Neorecormon^®^), darbepoetin alfa (B03XA02; Aranesp^®^), epoetin zeta (B03XA01; Retacrit^®^), and methoxypolyethyleneglycol-epoetin beta (B03XA03; Mircera^®^). Binocrit^®^, Abseamed^®^ and Retacrit^®^ are biosimilars of the reference product (Eprex^®^) (S1 Table), while all other ESAs are products still covered by the patent.

For each ESA dispensing, information about marketing authorization code (AIC) and brand name of the drug, dispensing date, number of dispensed packages of the drug were retrieved from electronic therapeutic plan as well as pharmacy dispending records.

### Data Analysis

Patients with ESA treatment (ESA users) were classified according to indication for use (CKD and cancer) and type of dispensed ESA (biosimilar, reference product and other ESAs covered by patent) at ID. Considering information collected in the electronic therapeutic plan, indication for use was mutually categorized as: (i) chronic kidney disease; (ii) cancer. In absence of an available electronic therapeutic plan, the indications for use were derived from other claims databases according to the algorithm described elsewhere [8].

At ID demographic and clinical characteristics (e.g. number of blood transfusions, hemoglobinemia and hematic level of creatinine, ferritin, folate, transferrin, vitamin B_12_, comorbidities including diabetes mellitus, hypertension, end stage renal disease, heart failure, and concomitant use of other iron preparations, folic acid, vitamin B_12_) of different ESA users has been evaluated. The strength of ESA therapy (defined as total number of defined daily dose (DDD) dispensed during the follow-up divided by days of follow-up) for biosimilar and other ESAs and the effect on Hb values after three months from the start of ESA treatment were investigated. In particular, the difference between follow-up and baseline Hb values, i.e. delta Hb (ΔHb), was calculated and ESA users were classified as non-responders (ΔHb≤0 g/dl), responders (0<ΔHb≤2 g/dl) and highly responders (ΔHb>2 g/dl).

### Sensitivity Analysis

In a sensitivity analysis the distribution of non-responders (ΔHb≤0 g/dl), responders (0<ΔHb≤2 g/dl) and highly responders (ΔHb>2 g/dl) was evaluating incident ESA user only at the first treatment episode.

### Statistical Analysis

Descriptive statistics were used to describe all examined variables.

In particular, results were presented as mean ± standard deviation (SD) or median with interquartile range depending on the underlying distribution for quantitative variables, and were summarized by absolute frequencies and percentages for categorical variables. Continuous and categorical variables were compared across groups according to type of ESA and indication for use at baseline using t-test or Chi-Square test (or Fisher's exact test when appropriate) for continuous and categorical variables, respectively.

For the multiple comparisons (reference product vs. biosimilar; other ESA vs. biosimilar), we applied Bonferroni’s correction, for which the significance alpha level 0.050 was divided by the number of the possible pairwise comparisons than can be performed with three groups; so the new “adjusted” significance level for this analysis is equal to 0.050 / 3 = 0.017.

In order to identify predictors of responsiveness of treatment with ESAs, univariate and multivariate ordinal logistic regression models, as Cumulative Proportional Odds Models [21–25] were performed stratifying by indication for use (CKD and cancer).

The dependent variable of the model was the responsiveness to ESA treatment, evaluated as the difference between follow-up (within 2^nd^ and 3^rd^ month after ESA treatment start) and baseline Hb values (ΔHb). It was expressed on three ordinal levels (non-responders: ΔHb≤0 g/dl; responders: 0>ΔHb≤2 g/dl; highly responders: ΔHb>2 g/dl).

As covariates we identified all the potential predictors of ESA responsiveness, including age, sex, strength of ESA treatment, type of ESA at ID (biosimilar, reference product or ESA covered by patent), year of treatment, first ever use of ESAs, co-morbidities (heart failure, hypertension, stage of CKD, dialysis), concomitant use of other drugs (e.g. folic acid, iron preparation, vitamin A and/or D, vitamin B_12_) and laboratory values (sideremia).

In the multivariate model, we included all the covariates that are well known to be clinically related to ESA responsiveness, irrespective of univariate analysis.

In order to evaluate if age, strength of ESA treatment, value of sideremia and baseline Hb value individually act as effect modifiers of the association between ESAs exposure and responsiveness to treatment, on the basis of clinical relevance of the investigated covariates, their interaction terms were individually included into the models and p-values were derived from the test for fixed effects.

For each covariate tested as possible predictor of responsiveness to treatment with ESAs, the ordered log-odds regression coefficients of the effect were reported along with 95% confidence interval (95% CI).

All statistical analyses were performed using SAS 9.2 (SAS Institute, Cary, NC) and SPSS/PC, Version 15 (SPSS Inc., Chicago, Illinois, USA). The significance level for all statistical tests was set at p-value < 0.05.

### Ethics Statement

This is an observational, retrospective, non-interventional study. In agreement with current national law, the study protocol was notified to the Ethical Committee of Academic Hospital of Treviso [26].

Written informed consent was not necessary as all data extracted from the database were fully anonymized prior to the authors receiving the data set.

## Results

**Fig 2:**
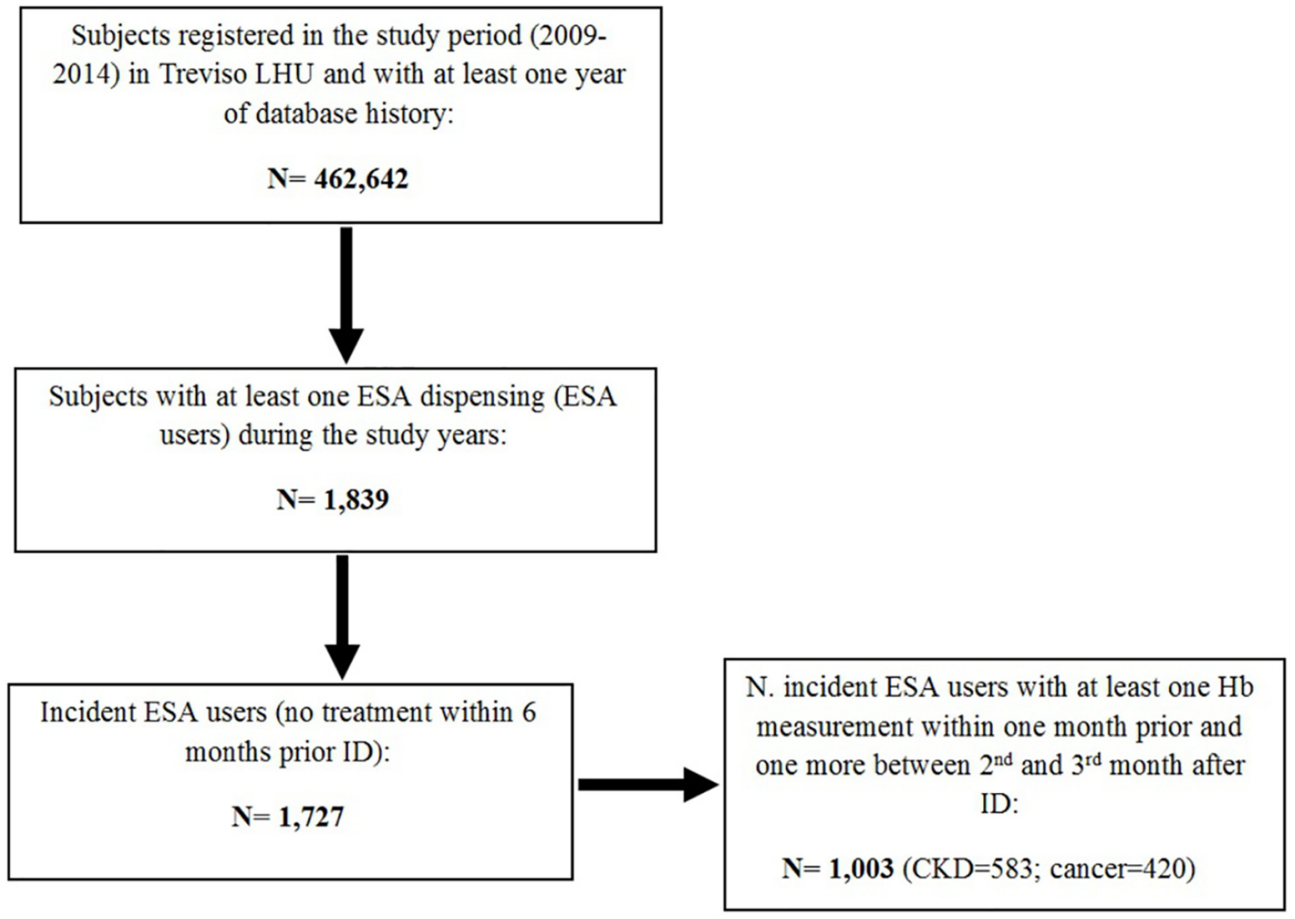
Inclusion in the study of incident ESA users from Treviso LHU. Index Date (ID) = date of ESA treatment start; LHU = Local Health Unit; ESA = Erythropoiesis-stimulating agents; Hb = Hemoglobin; CKD = Chronic Kidney Disease.

During the study period, on a total population of 462,642 subjects registered in Treviso LHU, 1,727 (0.4%) received at least one incident ESA dispensing during the years 2009–2014; of these, 1,003 (58.1%) ESA users had at least one baseline and one follow-up Hb measurement [CKD = 583 (58.1%); cancer = 420 (41.9%)] (Fig 2).

As shown in Table 1, ESAs were in general more frequently used by males in CKD and females in cancer, despite no statistically significant difference among various ESAs was observed. Regarding age distribution, ESA users with CKD starting a treatment with a biosimilar appear to be older (mean age ± SD: 80.4±8.7) than those starting with the reference product (mean age ± SD: 64.5±14.7, p-value = 0.055) or ESAs covered by patent (mean age ± SD: 66.0 ±16.1, p-value = 0.057), despite they did not reach statistical significance. CKD patients were more likely to use a biosimilar ESA (89.3%) for the first time than a reference product (77.1%) or an ESA covered by patent (78.3%). Blood transfusions before the starting treatment as well as switch after ESA treatment start were rare, without any statistically significant difference among ESA groups in both indications for use. On average, delta Hb after three months of ESAs treatment was 1.6±1.8 g/dl in CKD and 1.7±1.7 g/dl in cancer, with no difference across different types of ESAs.

**Table 1:** Demographic characteristics of incident ESA users, stratified by indication for use and type of ESA.

	CKD					Cancer				
	ESAs covered by patent N = 189 (%)	Reference product* N = 131 (%)	Biosimilar N = 263 (%)	P-value vs ESAs covered by patent^a^	P-value vs Reference product^a^	ESAs covered by patent N = 114 (%)	Reference product* N = 121 (%)	Biosimilar N = 185 (%)	P-value vs ESAs covered by patent^a^	P-value vs Reference product^a^
**Sex**										
Male	112 (59.3)	81 (61.8)	152 (57.8)	0.755	0.442	52 (45.6)	59 (48.8)	86 (46.5)	0.883	0.696
Female	77 (40.7)	50 (38.2)	111 (42.2)			62 (54.4)	62 (51.2)	99 (53.5)		
**Age ± SD**	66.0±16.1	64.5±14.7	80.4±8.7	0.057	0.055	68.1±12.2	64.3±13.7	68.7±11.8	0.748	0.211
**Age groups**										
<65	81 (42.8)	60 (45.8)	12 (4.6)	<0.001	<0.001	38 (33.3)	55 (45.5)	56 (30.3)	0.708	0.026
65–79	71 (37.6)	54 (41.2)	90 (34.2)			56 (49.1)	51 (42.1)	100 (54.0)		
≥ 80	37 (19.6)	17 (13.0)	161 (61.2)			20 (17.6)	15 (12.4)	29 (15.7)		
**First ever use of ESAs**^b^	148 (78.3)	101 (77.1)	235 (89.3)	0.001	0.001	98 (85.9)	103 (85.1)	163 (88.1)	0.589	0.449
**N. blood transfusions**^c^										
0	168 (88.9)	111 (84.7)	220 (83.6)	0.223	0.560	108 (94.7)	107 (88.4)	165 (89.2)	0.248	0.964
1	17 (9.0)	19 (14.5)	37 (14.1)			6 (5.3)	11 (9.1)	15 (8.1)		
2	3 (1.6)	1 (0.8)	6 (2.3)			-	2 (1.7)	4 (2.2)		
>2	1 (0.5)	-	-			-	1 (0.8)	1 (0.5)		
**Switch after ID**^d^	6 (3.1)	4 (3.0)	5 (1.9)	0.386	0.470	2 (1.7)	3 (2.5)	3 (1.6)	0.931	0.597
**Follow-up**^e^										
Mean±SD	70.3±16.1	70.4±16.2	68.6±16.4	0.470	0.489	74.0 ±16.4	75.5±14.3	73.9±15.7	0.966	0.526
**Difference between the follow-up Hb value and the baseline Hb values (Delta Hb) (g/dl)**										
Mean±SD	1.6±1.8	1.8±1.8	1.4±1.7	0.444	0.281	1.9±1.8	1.4±1.7	1.6±1.7	0.388	0.498
Median (q1-q3)	1.4 (0.4–2.7)	1.7 (0.5–3.1)	1.3 (0.2–2.5)			1.8 (0.5–3.0)	1.2 (0.5–2.3)	1.6 (0.5–2.5)		

SD = Standard Deviation; CKD = Chronic kidney disease; ESAs = Erythropoiesis-stimulating agents; ID = Index Date (starting ESA treatment date); Hb = Hemoglobin; q1-q3 = interquartile range;

*Reference product = Eprex;

^a^ p-value from two-sample t-test or Chi-Square test (or Fisher's exact test when appropriate) for continuous and categorical variables, respectively;

^b^ Number of ESA users without any ESA dispensing any time prior to ID;

^c^ Number of blood transfusions within six months prior to baseline Hb value;

^d^ Switch between starting ESA and another ESA was calculated within three months after ID;

^e^ Number of days between Index Date and the date of follow-up Hb value;

CKD patients receiving the reference product or an ESA covered by patent were more likely to be affected by end stage renal disease or hypertension and to use vitamin A/D than those using a biosimilar (who were more likely to be affected by heart failure). In general, no statistically significant difference in baseline demographic characteristics of cancer patients using different ESAs was observed. No statistically significant differences in laboratory values within three months prior to ID were found across users of different ESAs in both indications of use (Table 2).

**Table 2:** Distribution of comorbidities, concomitant drugs and labotratory values of incident ESA users, stratified by indication for use and type of ESA.

	CKD					Cancer				
	ESAs covered by patent N = 189 (%)	Reference product* N = 131 (%)	Biosimilar N = 263 (%)	P-value vs ESAs covered by patent^a^	P-value vs Reference product^a^	ESAs covered by patent N = 114 (%)	Reference product* N = 121 (%)	Biosimilar N = 185 (%)	P-value vs ESAs covered by patent^a^	P-value vs Reference product^a^
**Comorbidities**^b^										
Diabetes mellitus	72 (38.0)	48 (36.6)	105 (39.9)	0.694	0.529	23 (20.2)	27 (22.3)	45 (24.3)	0.406	0.685
End Stage Renal Disease (on dialysis)	32 (16.9)	37 (28.2)	18 (6.8)	0.000	<0.001	-	2 (1.6)	1 (0.5)	0.432	0.334
Hypertension	181 (95.8)	117 (89.3)	228 (86.7)	0.001	0.458	72 (63.1)	70 (57.8)	116 (62.7)	0.937	0.395
Heart failure	35 (18.5)	28 (21.4)	95 (36.1)	<0.001	0.003	5 (4.4)	7 (5.8)	8 (4.3)	0.979	0.563
**Concomitant drugs**^c^										
Iron preparation	40 (21.2)	24 (18.3)	38 (14.4)	0.062	0.320	7 (6.1)	6 (4.9)	10 (5.4)	0.789	0.863
Vitamin A and/or D	74 (39.1)	36 (27.5)	41(15.6)	<0.001	0.005	7 (6.1)	14 (11.6)	20 (10.8)	0.171	0.836
Vitamin B_12_	-	1 (0.8)	9 (3.4)	0.010	0.114	2 (1.7)	3 (2.5)	2 (1.1)	0.622	0.345
Folic acid	20 (10.6)	11 (8.4)	32 (12.2)	0.602	0.258	10 (8.8)	16 (13.2)	12 (6.5)	0.462	0.046
**Laboratory values: mean ±SD (n. ESAs users with ≥1 measurement within 3 months prior to ID)**^c^										
Albumin (g/dl; normal range: 3.5–5.5)	3.7±0.6 (116)	3.7±0.5 (101)	3.5±0.5 (120)	0.166	0.166	3.8±0.5 (42)	3.8±0.7 (46)	3.7±0.6 (72)	0.364	0.419
Folate (ng/ml; normal range: 2.7–17.0)	6.4±4.3 (30)	6.8±3.7 (23)	5.9±4.1 (92)	0.431	0.272	6.8±4.0 (21)	7.7±5.0 (18)	8.3±5.2 (24)	0.281	0.496
Ferritin (mcg/l; normal range: man: 60–300; woman: 30–150)	263.7±269.9 (105)	326.6±324.7 (78)	283.7±380.9 (150)	0.631	0.451	496.9±576.2 (29)	510.1±573.7 (31)	439.6±553.8 (43)	0.552	0.479
Transferrin (mg/dl; normal range: 200–400)	221.9±85.7 (17)	193.6±38.4 (10)	215.1±57.0 (34)	0.516	0.163	222.5±47.4 (15)	205.8±36.9 (13)	245.9±100.3 (9)	0.224	0.127
C-reactive protein (mg/dl; normal value: <0.5)	2.3±3.3 (112)	2.3±4.4 (96)	2.7±3.6 (154)	0.436	0.533	2.2±3.2 (48)	3.6±6.6 (48)	3.3±4.2 (94)	0.237	0.733
Creatinine (mg/dl; normal range:man:0.7–1.2; woman: 0.6–1.2)	3.5±2.3 (187)	2.9±1.9 (126)	2.7±1.5 (256)	0.149	0.483	0.9±0.4 (107)	1.1±0.9 (100)	1.0±0.5(163)	0.307	0.465
Parathyroid hormone (pg/ml; normal range:10–60)	272.6±245.8 (87)	206.8±220.2 (51)	212.8±191.6 (62)	0.219	0.834	33.0±25.6 (4)	47.5± 2.1 (2)	56.3±44.6 (9)	0.109	0.227
Potassium (mEq/l; normal range:3.6–5.0)	4.7±0.6 (183)	4.5±0.6 (124)	4.6±0.6 (250)	0.330	0.363	4.5±0.5 (82)	4.3±0.4 (80)	4.3±0.5 (137)	0.184	1.000
Vitamin B_12_ (ng/ml; normal range:300–900)	568.8±228.2 (28)	489.2±247.4 (22)	452.9±204.4 (97)	0.113	0.384	460.7±195.3 (16)	557.1±245.7 (25)	465.9±189.5 (20)	0.858	0.178
Sideremia (mcg/dl; range: man: 75–160; normal woman: 60–150)	59.3±29.8 (104)	57.4±29.7 (86)	59.3±50.5 (141)	1.000	0.720	63.7±46.4 (59)	72.2±42.3 (49)	69.2±52.9 (65)	0.518	0.680

SD = Standard Deviation; CKD = Chronic kidney disease; ESAs = Erythropoiesis-stimulating agents; ID = Index Date (starting ESA treatment date); q1-q3 = interquartile range;

*Reference product = Eprex;

^a^ p-value from two-sample t-test or Chi-Square test (or Fisher's exact test when appropriate) for continuous and categorical variables, respectively;

^b^ Co-morbidities have been evaluated any time prior to ID;

^c^ Concomitant drugs and laboratory values have been evaluated within three months prior to ID.

The significance level of the results did not change after applying the Bonferroni’s correction.

**Fig 3:**
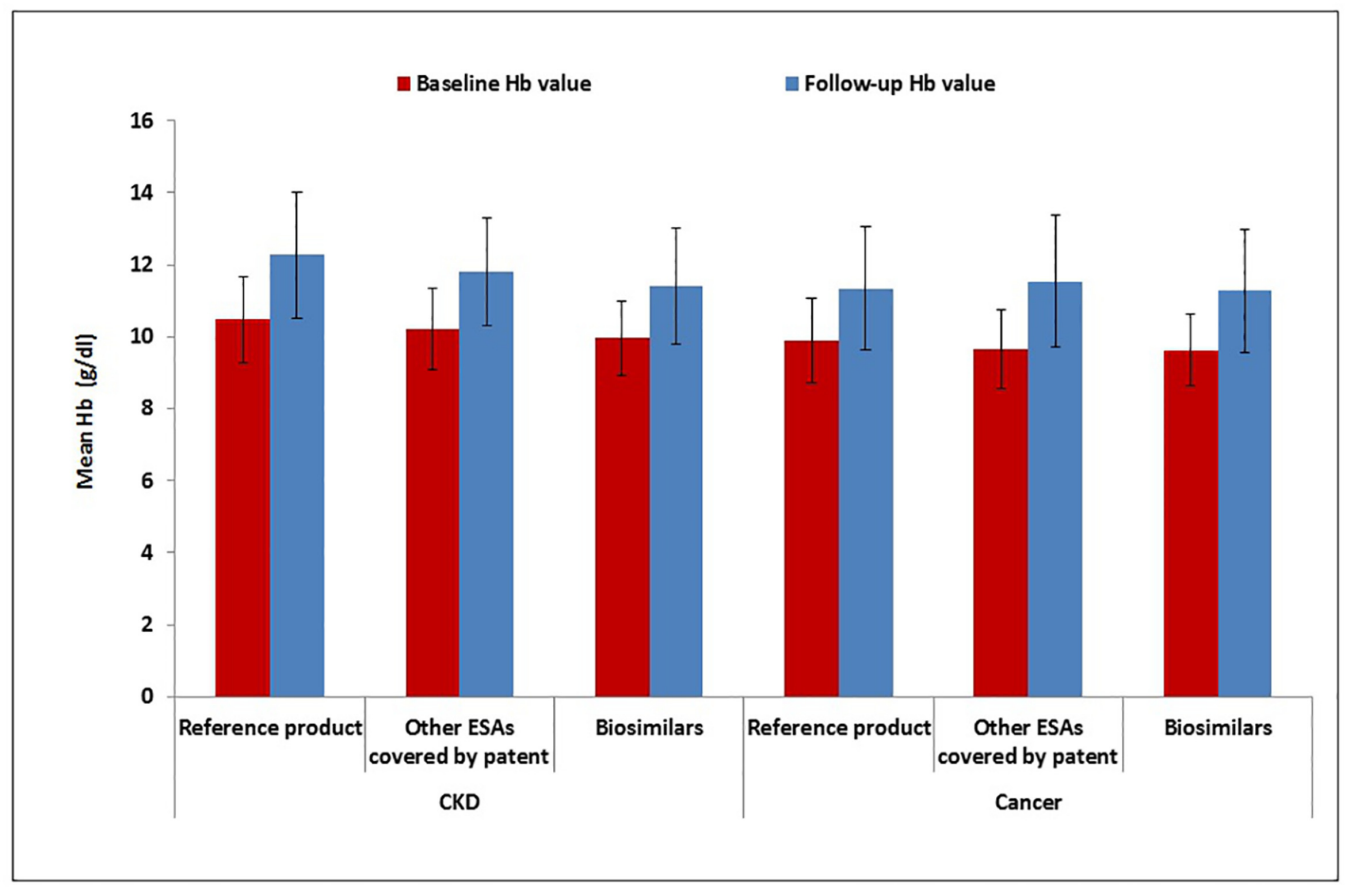
Effect of ESAs on Hb measurements within the follow-up Hb value from the start of the treatment, stratified by indication for use. Hb = hemoglobin; CKD = Chronic kidney disease; ESAs = Erythropoiesis-stimulating agents.

As shown in Fig 3, there was no statistically significant difference in mean baseline Hb value (g/dl) for biosimilars vs. reference product/other ESAs covered by patent in the two indications for use, separately (CKD: biosimilars: mean±SD = 9.9±1.0 g/dL; reference product: mean±SD = 10.4±1.2 g/dL; ESAs covered by patent: mean±SD = 10.2±1.1 g/dL; biosimilars vs reference product: p-value = 0.152; biosimilars vs ESAs covered by patent: p-value = 0.206; Cancer: biosimilars: mean±SD = 9.6±0.9 g/dL; reference product: mean±SD = 9.9±1.2 g/dL; ESAs covered by patent: mean±SD = 9.6±1.1 g/dL; biosimilars vs reference product: p-value = 0.256; biosimilars vs ESAs covered by patent: p-value = 1.000). The same held true for Hb value measured during the follow-up (CKD: biosimilars: mean±SD = 11.4±1.6 g/dL; reference product: mean±SD = 12.3±1.7 g/dL; ESAs covered by patent: mean±SD = 11.8±1.5 g/dL; biosimilars vs reference product: p-value = 0.124; biosimilars vs ESAs covered by patent: p-value = 0.224; Cancer: biosimilars: mean±SD = 11.3±1.7 g/dL; reference product: mean±SD = 11.3±1.7 g/dL; ESAs covered by patent: mean±SD = 11.5±1.8 g/dL; biosimilars vs reference product: p-value = 1.000; biosimilars vs ESAs covered by patent: p-value = 0.515).

**Fig 4:**
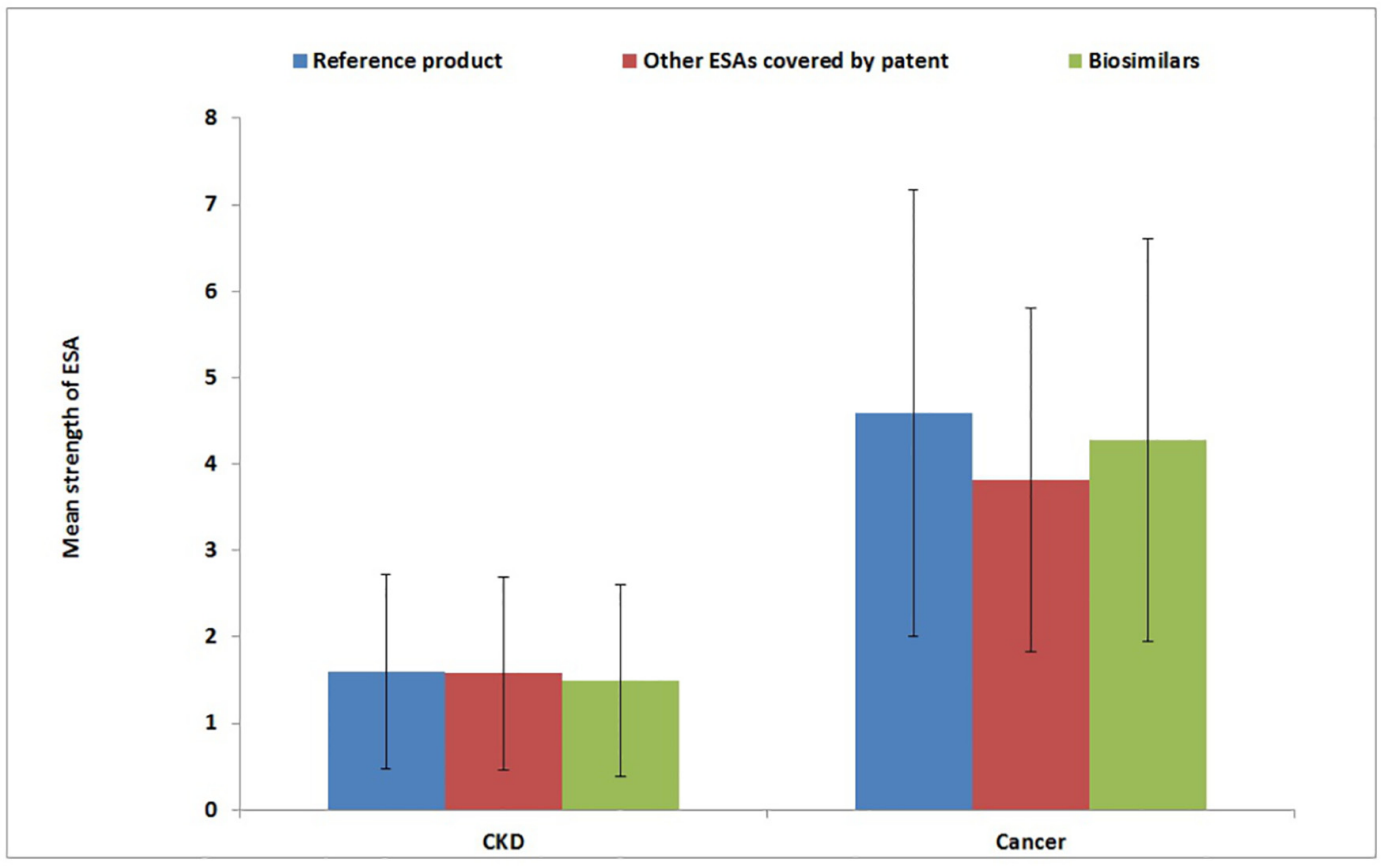
Mean strength of ESA treatment during the whole follow-up, stratified by indication for use and type of ESA. CKD = Chronic kidney disease; ESAs = Erythropoiesis-stimulating agents.

As shown in Fig 4, no statistically significant difference in strength of ESA treatment among different types of ESAs was found in either CKD (biosimilar: mean±SD = 1.5±1.1; reference product: mean±SD = 1.6±1.1; ESAs covered by patent: mean±SD = 1.6±1.1) and cancer (biosimilar: mean ± SD = 4.3±2.3; reference product: mean±SD = 4.6±2.6; ESAs covered by patent: mean ±SD = 3.8±2.0) patients, with much higher doses used to treat chemotherapy-induced than CKD-related anemia.

**Fig 5:**
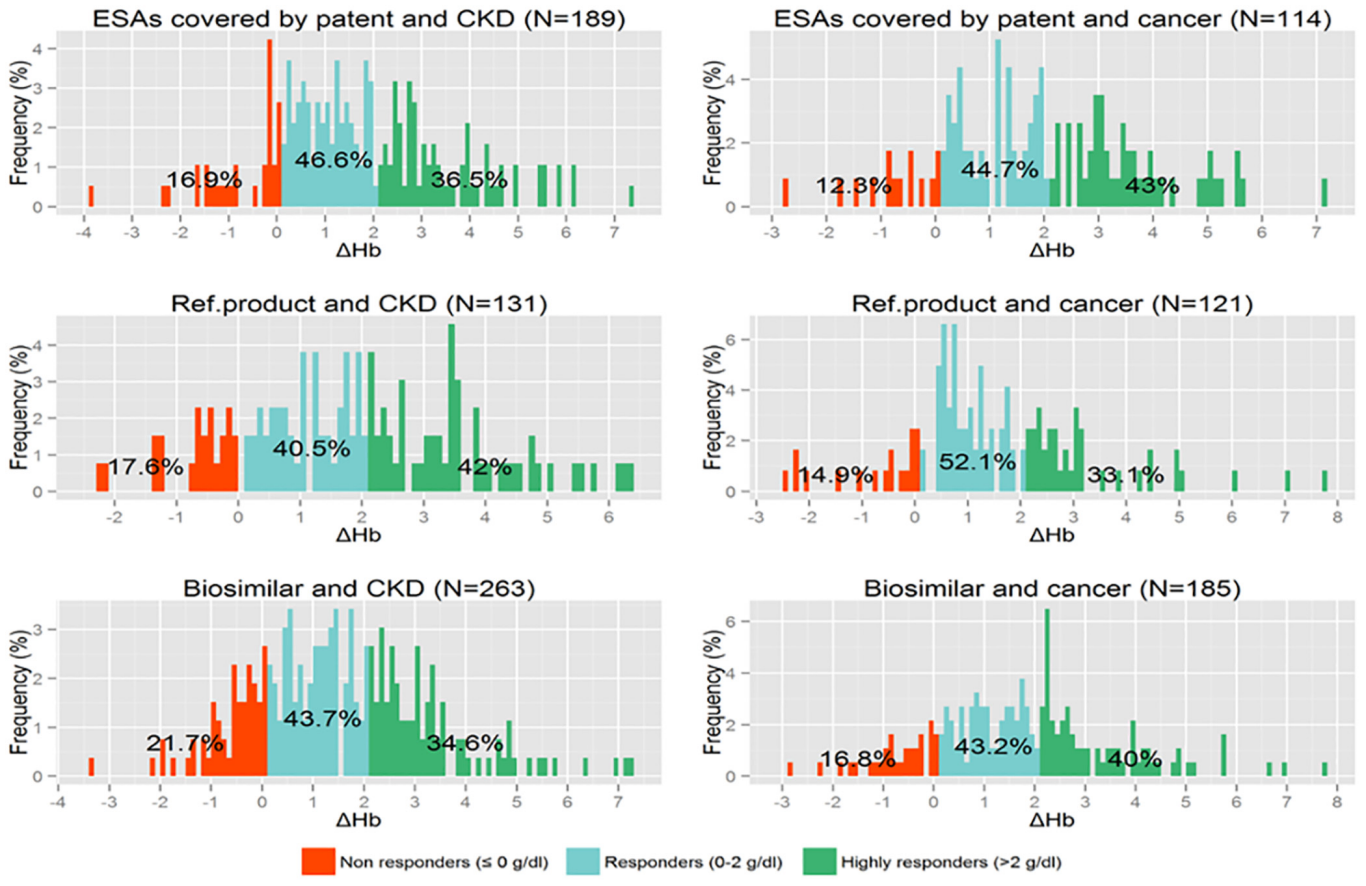
Responsiveness to ESA treatment, assessed as delta Hb (ΔHb) between follow-up and baseline Hb value, stratified by indication for use and type of ESA. Hb = hemoglobin; CKD = Chronic kidney disease; ESAs = Erythropoiesis-stimulating agents; Ref. Product = Reference Product.

As observed in Fig 5, around 20% of ESA users were non-responders in both CKD and cancer. No differences across types of ESAs users were found in terms of distribution of responders and highly responders in both indications for use.

In a sensitivity analysis we excluded ESAs users who were selected multiple times as study patient and we did not find any difference in comparison to the main analysis.

As output of the univariate ordinal logistic regression model, the strength of treatment (coefficient: 0.42; IC95%: 0.26/0.57; P-value<0.001) and the previous use of iron preparations (coefficient: 0.45; IC95%: 0.05/0.86; P-value = 0.028) were statistically associated with an increase of responsiveness, while first ever use of ESAs (coefficient: -0.43; IC95%: -0.83/-0.21; P-value = 0.039) and a previous history of heart failure (coefficient: -0.39; IC95%: -0.73/-0.51; P-value = 0.024) were associated with a decrease of responsiveness in CKD patients. A previous use of vitamin B_12_ (coefficient: -1.45; IC95%: -2.87/-0.03; P-value = 0.046) seemed associated with a decrease of responsiveness in cancer patients. Type of ESA was not associated to responsiveness in both indications of use.

After adjusting for all potential clinical confounders, previous history of heart failure, first ever use of ESAs and previous use of iron preparation in CKD and previous use of vitamin B12 in cancer patients lost the statistical significance; type of ESA did not remain a predictor of responsiveness to treatment for both indications for use. On the other hand, previous history of hypertension and strength of ESA treatment were associated with an increase of responsiveness to ESA treatment in CKD, while sex (male) was negatively associated with responsiveness to ESA treatment in cancer (Table 3).

**Table 3:** Multivariate Ordinal Logistic Regression Model for responsiveness to first three months of ESA therapy.

	CKD		Cancer	
	Coefficient (95% CI)	P-value	Coefficient (95% CI)	P-value
**Variables**				
Sex (Males)	-0.14 (-0.58/0.30)	0.525	-0.92 (-1.54/-0.31)	0.003
Age	-0.05 (-0.02/0.01)	0.574	0.003 (-0.02/0.03)	0.827
**Type of ESA**				
Biosimilar	**Comparator**			
Reference Product	0.25 (-0.37/0.87)	0.435	-0.07 (-0.85/0.71)	0.859
ESAs covered by patent	-0.29 (-1.02/0.44)	0.436	-0.07 (-0.84/0.70)	0.867
**First ever use of ESA treatment**	0.35 (-0.29/0.99)	0.285	0.52 (-0.57/1.60)	0.351
**Strength of ESA treatment**	0.35 (0.15/0.56)	<0.001	0.02 (-0.12/0.16)	0.815
**Year of treatment**	-0.06 (-0.28/0.15)	0.571	-0.17 (-0.44/0.10)	0.209
**Comorbidities**				
Heart failure	-0.40 (-0.90/0.10)	0.120	-0.75 (-2.04/0.54)	0.253
Hypertension	1.04 (0.28/1.80)	0.007	0.24 (-0.45/0.93)	0.494
Dialysis	-0.55 (-1.36/0.26)	0.181	-	-
**CKD stage**				
1–3	-0.10 (-0.81/0.61)	0.786	-	-
4	-0.31 (-1.02/0.40)	0.391	-	-
5 and dialysis	**Comparator**			
**Concomitant drugs**				
Iron preparation	0.23 (-0.35/0.81)	0.436	0.21 (-0.79/1.21)	0.678
Vitamin B_12_	-0.33 (-1.77/1.11)	0.653	-1.86 (-3.91/0.18)	0.074
Vitamin A and/or D	-0.11 (-0.64/0.42)	0.675	0.15 (-1.22/1.53)	0.828
Folic acid	0.09 (-0.61/0.80)	0.791	0.53 (-0.58/1.64)	0.348
**Laboratory value**				
Sideremia	0.002 (-0.003/0.007)	0.466	0.002 (-0.004/0.008)	0.544

CKD = Chronic kidney disease; ESAs = Erythropoiesis-stimulating agents; CI = Confidence Interval.

The association between exposure to different ESAs and responsiveness to the treatment was not significantly modified by the age, sideremia or baseline Hb value in CKD and cancer. Only the strength of treatment is an effect modifier of the responsiveness in CKD ESAs users (p-value<0.001).

## Discussion

To our knowledge this is the first population based-study which compared the effects of biosimilar vs. reference product and vs. other ESAs covered by patent on haemoglobinemia in both CKD and cancer patients using real world data.

Overall, our results suggested that the effect on Hb values after three months of treatment was comparable among various types of ESAs both in CKD or cancer (patients.

These findings were in line with pre-marketing randomized clinical trials (RCTs), in which Hb response was defined as an increase in Hb concentration of ≥ 2.0 g/dl from the mean value from baseline to weeks 5–12 of the study [14] or end of week 13 after the treatment start [27], and post-marketing studies. Pre-marketing RCTs investigated mostly the comparative therapeutic efficacy and safety of reference product and biosimilar ESAs in the treatment of CKD-related anemia, highlighting no differences in achieving the target Hb value [9–11]. The first two clinical trials compared the efficacy and safety of epoetin zeta (biosimilar of epoetin alfa) vs. epoetin alfa, administered intravenously [9, 10], achieving a similar target of Hb value (epoetin zeta: 11.6±1.3 g/dL; epoetin alfa: 11.6±1.4 g/dL) over the last 4 weeks, on a total of 24 weeks of treatment [10]. A study on 462 anemic patients with end stage renal failure on chronic hemodialysis demonstrated the therapeutic equivalence in maintaining the Hb concentration of epoetin zeta and epoetin alfa administered subcutaneously [11]. So all these RCTs suggested that epoetin zeta is a clinically equivalent, well-tolerated alternative to epoetin alfa in patients with renal anemia.

To date, only one randomized, multicentre, double blind study was performed to evaluate the efficacy and safety of a biosimilar of epoetin alfa in the treatment of chemotherapy-induced anemia. Seventy-four patients were allocated to treatment with biosimilar of epoetin alfa and 40 patients were allocated to treatment with reference product ESAs. [14].

On average, a small sample of patients was eligible in each premarketing RCT of biosimilar ESAs both in CKD (460 patients) and cancer (114 patients). On the contrary, in our study, we included more than 1,000 patients affected by CKD (N = 583) or cancer (N = 420) from a general population from Northern Italy.

Our findings were also confirmed by a multicenter retrospective study evaluating the comparative effectiveness of biosimilar of epoetin alfa vs. darbepoetin alfa after about four weeks of treatment in cancer patients from a community-based single German center (mean baseline Hb value (g/dL): biosimilar: 9.8; darbepoetin alfa: 9.9; mean Hb at end of ESA treatment (g/dL): biosimilar: 11.9; darbepoetin alfa: 11.9). Similar results were found in a large oncology department of a Spanish hospital with biosimilar and darbepoetin alfa (mean baseline Hb value (g/dL): 9.3; mean Hb at end of ESA treatment (g/dL): 10.8) [18].

More importantly, we did not find any statistically significant difference between biosimilars and reference product/ESAs covered by patent in terms of responsiveness, despite a comparable prescribed cumulative dose of drug during the observation period. This finding is in contrast with the results reported in a recently published research letter of Minutolo R. et al. showing that switching from ESA originator to biosimilar may require higher doses of biosimilar ESA to maintain Hb levels [28]. Several differences may explain the differences observed in ours and Minutolo’s study: a) we investigated all naïve users of different ESAs while Minutolo et al. analysed only the switchers from originator to biosimilar. As renal functionality declines progressively thus requiring generally higher doses of all ESAs over time to achieve target Hb, it may be more appropriate to investigate all possible switch between biosimilars and reference product/other ESAs covered by patent and vice versa; b) we conducted a population-based study while Minutolo et al. conducted a multi-center study where patients with stable ESA originator doses were previously selected, thus preventing the generalization of the results to the whole spectrum of the hemodialysis population.

Importantly, we found that 15–20% of ESA users were not responders, with no significant differences across various ESAs. So, efforts should be put to find factors associated with poor response in ESA-resistant patients, irrespective of the ESA being prescribed.

### Strengths and Limitations

The main strength of this population-based study is the possibility to explore Hb value changes as a result of all available ESAs using real world data on a population level for more than 1,000 ESA users.

Most of the previous RCTs were conducted only considering CKD patients, while our study compared the effectiveness of biosimilar and reference product/ ESAs covered by patent on haemoglobinemia both in CKD and in cancer patients.

Availability of electronic therapeutic plans provides information on the exact brand name, dosing regimen, and indication for use. Moreover, availability of laboratory values provides clinically relevant information regarding some resistance-factors to ESA treatments (e.g. C-reactive protein, high levels of parathyroid hormone, low levels of vitamin B12, and folate).

Some limitations warrant caution. Some ESA dispensing as well as concomitant drugs (i.e. iron preparations) might not have been captured by the LHU database, as drugs can be initially dispensed directly in the hospital. However, it is unlikely that this limitation affected the study results as misclassification is expected to be minimal and non-differential across different types of ESAs.

## Conclusions

No difference on the short-term effects on hemoglobinemia among users of biosimilar ESAs or reference product/ESAs covered by patent was observed in a general population from Northern Italy, despite a comparable dispensed dose of different ESAs during the first three months of treatment.

## Supporting Information

S1 Table Available ESAs in Treviso LHU database during the study years. ATC = Anatomical Therapeutic Chemical (ATC) Classification System; CKD = Chronic kidney disease. Note: some study drugs (Nespo: darbepoetin alfa; Abseamed, Globuren: epoetin alfa; Eporatio: epoetin theta) were marketed in Italy, but they were not available in Treviso database.
(PDF)

## References

[1] Aapro Matti S and Link Hartmut. September 2007 Update on EORTC Guidelines and Anemia Management with Erythropoiesis-Stimulating Agents. The Oncologist. 2008; 13:33–6. 10.1634/theoncologist.13-S3-33 18458123

[2] Determinazione AIFA. Aggiornamento del Piano terapeutico AIFA per prescrizione SSN di Eritropoietine (ex Nota 12). Available: http://www.agenziafarmaco.gov.it/sites/default/files/2010-07-29_determina_aggiornamento_template_nota_12_g.u._75_del_31-03-2009.pdf.

[3] Zuniga L, Calvo B. Biosimilars—the way forward. Hosp Pharm Europe. 2010; 50:33–4.

[4] Mellstedt H. The future of biosimilars. Hosp Pharm Europe. 2010;49: 33–34.

[5] Genazzani AA, Biggio G, Caputi AP, Del Tacca M, Drago F, Fantozzi R, et al Biosimilar drugs: concerns and opportunities. Biodrugs. 2007; 21:351–6. 18020619 10.2165/00063030-200721060-00003

[6] European Medicines Agency. Committee for Medicinal Products 569 for Human Use (CHMP): guideline on similar biological 570 medicinal products containing biotechnology-derived proteins as active substance: non-clinical and clinical issues. Available: http://www.ema.europa.eu/docs/en_GB/document_library/Scientific_guideline/2013/06/WC500144124.pdf.

[7] Loiacono C, Sgroi C, Coppolino S, Cannata A, Ferrara R, Arcoraci V, et al How much are biosimilars used in southern Italy? a retrospective analysis of epoetin utilization in the local health unit of Messina in the years 2010–2011. BioDrugs. 2012 4 1; 26(2):113–20. 10.2165/11630770-000000000-00000 22385406

[8] Ingrasciotta Y, Giorgianni F, Bolcato J, Chinellato A, Pirolo R, Tari DU, et al How Much Are Biosimilars Used in Clinical Practice? A Retrospective Italian Population-Based Study of Erythropoiesis-Stimulating Agents in the Years 2009–2013. BioDrugs. 2015 7 14. 10.1007/s40259-015-0132-7 PMC4561997 26169209

[9] Wizemann V, Rutkowski B, Baldamus C, Scigalla P, Koytchev R, Epoetin Zeta Study Group. Comparison of the therapeutic effects of epoetin zeta to epoetin alfa in the maintenance phase of renal anaemia treatment. Curr Med Res Opin. 2008; 24(3):625–37. 10.1185/030079908X273264 18208642

[10] Krivoshiev S, Todorov VV, Manitius J, Czekalski S, Scigalla P, Koytchev R, et al Comparison of the therapeutic effects of epoetin zeta and epoetin alpha in the correction of renal anaemia. Curr Med Res Opin. 2008; 24(5):1407–15. 10.1185/030079908X297402 18394266

[11] Krivoshiev S, Wizemann V, Czekalski S, Schiller A, Pljesa S, Wolf-Pflugmann M, et al Therapeutic Equivalence of Epoetin Zeta and Alfa, Administered Subcutaneously, for Maintenance Treatment of Renal Anemia. Adv Ther (2010) 27(2):105–117. 10.1007/s12325-010-0012-y 20369312

[12] Haag-Weber M, Vetter A, Thyroff-Friesinger U. Therapeutic equivalence, long-term efficacy and safety of HX575 in the treatment of anemia in chronic renal failure patients receiving hemodialysis. Clin Nephrol. 2009; 72(5):380–90. 19863881

[13] Palmer SC, Saglimbene V, Mavridis D, Salanti G, Craig JC, Tonelli M, et al Erythropoiesis-stimulating agents for anaemia in adults with chronic kidney disease: a network meta-analysis. Cochrane Database Syst Rev. 2014; 12:CD010590 10.1002/14651858.CD010590.pub2 25486075 PMC6885065

[14] Weigang-Köhler K, Vetter A, Thyroff-Friesinger U. HX575, recombinant human epoetin alfa, for the treatment of chemotherapy-associated symptomatic anaemia in patients with solid tumours. Onkologie. 2009 4;32(4):168–74. 10.1159/000200783 Epub 2009 Mar 6. 19372711

[15] Michallet M, Luporsi E, Soubeyran P, Amar NA, Boulanger V, Carreiro M, et al BiOsimilaRs in the management of anaemia secondary to chemotherapy in HaEmatology and Oncology: results of the ORHEO observational study. BMC Cancer. 2014; 14:503 10.1186/1471-2407-14-503 25011615 PMC4227033

[16] Bustos A, Álvarez R, Aramburo PM, Carabantes F, Díaz N, Florián J, et al Evaluation of clinical use and effectiveness of darbepoetin alfa in cancer patients with chemotherapy-induced anemia. Curr Med Res Opin. 2012; 28(1):57–67. 10.1185/03007995.2011.639352 22070513

[17] Kerkhofs L, Boschetti G, Lugini A, Stanculeanu DL, Palomo AG. Use of biosimilar epoetin to increase hemoglobin levels in patients with chemotherapy-induced anemia: real-life clinical experience. Future Oncol. 2012; 8(6):751–6. 10.2217/fon.12.39 22443466

[18] Garzotto AR, Heine O, Turner M, Rebollo Laserna F, Lorenz A. Erythropoiesis-stimulating agents for the treatment of chemotherapy-induced anemia: comparisons from real-world clinical experience. Journal of Blood Medicine. 2014; 5: 43–48. 10.2147/JBM.S57887 24855398 PMC4011805

[19] Agenzia Italiana del Farmaco. Position Paper sui farmaci biosimilari. Available: http://www.agenziafarmaco.gov.it/it/content/position-paper. Accessed 2 Feb 2 2014.

[20] Morkeberg J, Lundby C, Nissen-Lie G, Nielsen TK, Hemmersbach P, Damsgaard R. Detection of darbepoetin alfa misuse in urine and blood: preliminary investigation. Med Sci Sports Exerc. 2007; 39: 1742–7. 17909401 10.1249/mss.0b013e31811e9d55

[21] Ananth CV, Kleinbaum D G. Regression models for ordinal responses: A review of methods and applications. International Journal of Epidemiology. 1997; 26, 1323–1333. 9447413 10.1093/ije/26.6.1323

[22] Brant R. Assessing proportionality in the proportional odds model for ordinal logistic regression. Biometrics. 1990; 46, 1171–1178. 2085632

[23] O’Connell A A. Logistic regression models for ordinal response variables. Thousand Oaks, CA: SAGE 2006.

[24] Agresti A. 2010 Analysis of Ordinal Categorical Data, Wiley, 2nd ed.

[25] Liu I, and Agresti A. 2005 The analysis of ordered categorical data: An overview and a survey of recent developments (with discussion). Test 14: 1–73.

[26] Ministero della Salute, Agenzia Italiana del Farmaco. Circolare AIFA del 3 agosto 2007. Linee guida per la classificazione e conduzione degli studi osservazionali sui farmaci. Available: http://xoomer.virgilio.it/pgiuff/osservazionali.pdf.

[27] Haag-Weber M, Eckardt KU, Hörl WH, Roger SD, Vetter A, Roth K. Safety, immunogenicity and efficacy of subcutaneous biosimilar epoetin-α (HX575) in non-dialysis patients with renal anemia: a multi-center, randomized, double-blind study. Clin Nephrol. 2012 1;77(1):8–17. 22185963 10.5414/cn107304

[28] Minutolo R, Borzumati M, Sposini S, Abaterusso C, Carraro G, Santoboni A, et al Dosing Penalty of Erythropoiesis-Stimulating Agents After Switching From Originator to Biosimilar Preparations in Stable Hemodialysis Patients. Am J Kidney Dis. 2016 2 13. 10.1053/j.ajkd.2016.01.011 26879099

