# Supplementary material for: Comparative Effectiveness of Biosimilar, Reference Product and Other Erythropoiesis-Stimulating Agents (ESAs) Still Covered by Patent in Chronic Kidney Disease and Cancer Patients: An Italian Population-Based Study
Source: PLoS One. 2016 May 17;11(5):e0155805. doi: 10.1371/journal.pone.0155805 (PMC4871488; doi:10.1371/journal.pone.0155805)
Supplement: S1 Table — ATC = Anatomical Therapeutic Chemical (ATC) Classification System; CKD = Chronic kidney disease. Note: some study drugs (Nespo: darbepoetin alfa; Abseamed, Globuren: epoetin alfa; Eporatio: epoetin theta) were marketed in Italy, but they were not available in Treviso database. (PDF) [file pone.0155805.s001.pdf]

S1 Table. Available ESAs in Treviso LHU database during the study years.

Brand name	Molecule	ATC	Type of ESA	Indication for use
**Aranesp**	Darbepoetin alfa	B03XA02	Drug covered by patent	Anemia due to CKD in adult/pediatric patients; Anemia induced by anticancer chemotherapy in adult patients
**Eprex**	Epoetin alfa	B03XA01	Reference product	Anemia due to CKD in adult/pediatric patients; Anemia induced by anticancer chemotherapy in adult patients
**Binocrit**	Epoetin alfa	B03XA01	Biosimilar	Anemia due to CKD in adult/pediatric patients; Anemia induced by anticancer chemotherapy in adult patients
**Neorecormon**	Epoetin beta	B03XA01	Drug covered by patent	Anemia due to CKD in adult/pediatric patients; Anemia induced by anticancer chemotherapy in adult patients
**Retacrit**	Epoetin zeta	B03XA01	Biosimilar	Anemia due to CKD in adult/pediatric patients; Anemia induced by anticancer chemotherapy in adult patients
**Mircera**	Methoxypolyethylene glycol-epoetin beta	B03XA03	Drug covered by patent	Anemia due to CKD in adults
